# *T*_1_ Relaxation Measurement of *Ex-Vivo* Breast Cancer Tissues at Ultralow Magnetic Fields

**DOI:** 10.1155/2015/385428

**Published:** 2015-01-29

**Authors:** Seong-Joo Lee, Jeong Hyun Shim, Kiwoong Kim, Seong-min Hwang, Kwon Kyu Yu, Sanghyun Lim, Jae Ho Han, Hyunee Yim, Jang-Hee Kim, Yong Sik Jung, Ku Sang Kim

**Affiliations:** ^1^Center for Biosignals, Korea Research Institute of Standards and Science (KRISS), 267 Gajeong-ro, Yuseong-gu, Daejeon 305-340, Republic of Korea; ^2^Department of Medical Physics, University of Science and Technology (UST), 217 Gajeong-ro, Yuseong-gu, Daejeon 305-333, Republic of Korea; ^3^Department of Pathology, Ajou University School of Medicine, 164 World Cup-ro, Yeongtong-gu, Suwon 443-380, Republic of Korea; ^4^Department of Surgery, Ajou University School of Medicine, 164 World Cup-ro, Yeongtong-gu, Suwon 443-380, Republic of Korea

## Abstract

We investigated *T*_1_ relaxations of *ex-vivo* cancer tissues at low magnetic fields in order to check the possibility of achieving a *T*_1_ contrast higher than those obtained at high fields. The *T*_1_ relaxations of fifteen pairs (normal and cancerous) of breast tissue samples were measured at three magnetic fields, 37, 62, and 122 *μ*T, using our superconducting quantum interference device-based ultralow field nuclear magnetic resonance setup, optimally developed for *ex-vivo* tissue studies. A signal reconstruction based on Bayesian statistics for noise reduction was exploited to overcome the low signal-to-noise ratio. The ductal and lobular-type tissues did not exhibit meaningful *T*_1_ contrast values between normal and cancerous tissues at the three different fields. On the other hand, an enhanced *T*_1_ contrast was obtained for the mucinous cancer tissue.

## 1. Introduction

Breast cancer is one of the leading causes of cancer mortality in women. Undoubtedly, early diagnosis of breast cancer is beneficial to patients in terms of lowering mortality and enabling breast-conserving surgery. The mammogram is widely used for screening; however, it has the limitation of low sensitivity to dense breast tissue [1], and the method of breast compression necessary for this procedure may result in patients experiencing discomfort and pain. In particular, a significant population in some Asian countries has insufficient breast volume to respond to mammographic examination properly, and additionally, these patients feel uneasy with this examination as a result of cultural reluctance. In addition to mammograms, magnetic resonance imaging (MRI) is considered a supplementary scanning technique, but its role in screening is debated [2, 3]. The American Cancer Society recommends MRIs only for people who have a high life-time risk (>20%) due to family history [4]. In order to obtain an improved magnetic resonance (MR) image of breast tumors, patients may have to take contrast agents, which, in principle, are preferably avoided because of potential side effects. Therefore, the development of a new modality that provides confirmative imaging for early diagnosis of breast cancer still needs to be pursued in biomedical research.

In general, the discrimination between tumors and healthy tissue using MRI primarily relies on differences in the water density of these tissues. In the early stages of cancer, however, the water density in the cancerous tissue is expected to be similar to that of normal tissue. To achieve a *T*_1_-contrast MR image of the cancerous tissues, magnetic field strength should be adjusted to elaborate the difference in the spin-lattice relaxation times (*T*_1_). Field-cycling relaxometry studies [5–8] have been performed on various biological mediums; the human brain have shown that the largest *T*_1_ contrast is accessible at low magnetic fields, that is, at less than 300 *μ*T [9]. An agarose gel solution has shown drastically enhanced *T*_1_ contrasts at magnetic fields of less than 100 *μ*T [10, 11]. The *T*_1_-weighted imaging of the tissue of a rat tumor was obtained at approximately 100 *μ*T [12]. Recent *T*_1_ measurements of prostate tissue at 132 *μ*T have revealed that the *T*_1_ contrast has a linear dependence on the ratio of the cancer volume in the tissue samples [13]. Inspired by these previous studies, we focus on* ex-vivo* relaxometry of breast cancer tissue samples at low magnetic field conditions here. Since the mammary gland is functionally and qualitatively similar to prostate tissue, an enhanced *T*_1_ contrast of breast cancer tissue can be expected at such low fields. Our experimental procedure is conducted on a homebuilt ultralow field (ULF)-nuclear magnetic resonance (NMR) setup [14–19] using superconducting quantum interference device (SQUID) to sense weak NMR signals, owing to the low static magnetic field.

SQUID-based microtesla NMR has paved the way for new biomedical measurements under low magnetic field conditions, for example, chemical analysis from a *J*-coupling spectrum [20], simultaneous proton density imaging with magnetoencephalography [21, 22], direct neutral current imaging [23–25], brainwave magnetic resonance [18], and heart magnetic resonance [26]. All of these measurements are difficult to achieve with conventional high-field NMR/MRIs. The present study's aim, to achieve confirmative MR imaging of cancer tissues,* in-vivo* and contrast agent free, will certainly be one of the most fruitful applications of low field MRI.

In this study, we measured the *T*_1_ contrasts of breast cancer tissues at three different low fields, 37, 62, and 122 *μ*T. The *T*_1_ trend variation may give us a relaxometric fingerprint to discriminate between breast cancer and healthy tissue, either by estimating an optimal magnetic field or by comparing the slope of the *T*_1_ variation along the different external magnetic fields. Since the tissue volumes were small (approximately 1 cm^3^), relatively low signal-to-noise ratios (SNR) were obtained. Hence, we used a signal reconstruction method based on Bayesian statistics for noise removal, and reliably separated signal amplitudes from noise. According to the model for relaxation in a multiphase system [27], provided that the exchange rate is faster than the relaxation rate (1/*T*_1_), the relaxation rate has a linear dependence on the volume ratio, and the coefficient of the linearity provides the *T*_1_ contrast between the media. From the same analysis of our data, the *T*_1_ contrasts at three magnetic fields failed to exhibit a meaningful relevance to the volume ratio of breast cancer in tissue. One result worth noting is that mucinous carcinoma tissue can be distinguished clearly from other tissue, because of its high water density and long *T*_1_ relaxation time. Possible implications of our results and further study will be discussed.

## 2. Methods

### 2.1. Sample Treatment

In this study, fifteen pairs of breast tissue specimens were investigated. Each pair consists of nominal tumor and normal tissues. All the tissue samples were obtained from total mastectomy specimens in Ajou University Hospital (Suwon, Korea). The tumor tissue samples were found to include various cancer types: ten invasive ductal carcinomas, one ductal carcinoma* in situ*, one mixed ductal and lobular carcinoma, and one mucinous carcinoma. Apart from the mucinous case, discrimination between cancer types was not the concern of this study. This research was approved by the Institutional Review Board of Ajou University Hospital.

After gross examination of each mastectomy specimen, representative tumor and normal tissue samples with volumes of approximately 1 cm^3^ were prepared and then immediately inserted into a liquid nitrogen (LN2) tank, where they were stored until the NMR experiments were conducted, following Huang et al. [8]. Prior to the *T*_1_ measurements, the tissues were thawed in the ambient atmosphere and temperature. To avoid dehydration after thawing, they were wrapped in cling film.

The specimens were formalin-fixed after the NMR measurements were complete. The paired tumor and normal tissue samples were routinely processed and stained with hematoxylin-eosin for histologic examination. From each tissue, a single slide was prepared via sectioning and examined with a microscope. The proportion ratios of cancer cells were measured by mapping the slides on a square grid in millimeter.

### 2.2. Experimental Setup

**Figure 1:**
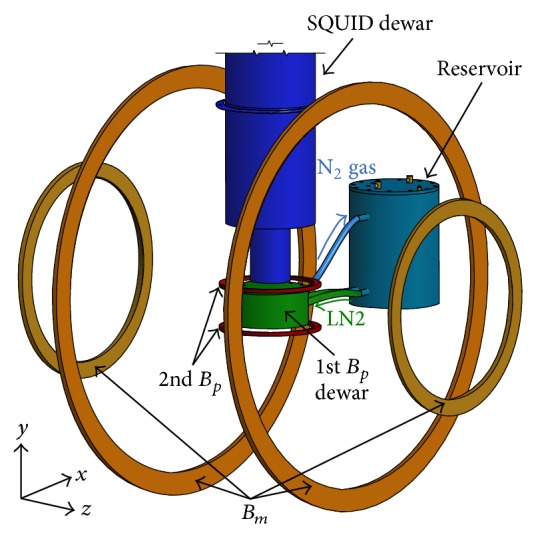
Schematic diagram of the experimental apparatus for the microtesla NMR experiment. The axes of the 1st and 2nd *B*_*p*_ coils are parallel. However, these are perpendicular to the axis of the *B*_*m*_ coil. The 1st *B*_*p*_ coil is a liquid nitrogen (LN2)-cooled pancake-type coil. The LN2 flowed continuously from the reservoir to the 1st *B*_*p*_ dewar through two tubes at the bottom and then the nitrogen gas flowed back to the reservoir through the upper tube.

Figure 1 shows the experimental setup for the microtesla NMR experiment. All equipment was mounted inside a magnetically shielded room (MSR), which was specially designed for ULF-NMR/MRI [18]. A dc-SQUID (Supracon AG) was used as a NMR signal detector for the ULF-NMR system. The dc-SQUID sensor was shielded with two different cans. The inner shield is a superconducting Nb cast can with 99.9% purity. The outer shield is a superconducting lead can with 99.5% purity. The pickup coil is a second-order gradiometer, which is made of a 125 *μ*m Nb wire with a 50 mm baseline and a diameter of 65 mm. The dc-SQUID with pickup coil was additionally wrapped with an aluminum-coated Mylar film to prevent the SQUID system from being influenced by ambient RF noise. The environmental noise of our system inside the MSR measured by the dc-SQUID sensor with second-order gradiometric pickup coil is about 2.3 fT/Hz^1/2^ at 100 Hz.

Since magnetization in the microtesla region is insufficient to produce the NMR signal in the ULF-NMR, a strong magnetic field (*B*_*p*_) for prepolarizing the nuclear magnetization must be applied prior to NMR signal acquisition. The *B*_*p*_ strength is in tens of mT. The free precession of the nuclear magnetization is then recorded under a measurement field (*B*_*m*_) in the *μ*T region. A double Helmholtz coil [28] was used to produce a high degree of homogeneity in the *B*_*m*_. The *B*_*m*_ strength at the sample space was about 4.93 *μ*T, which corresponds to a proton NMR frequency of about 210 Hz.

Producing a high *B*_*p*_ leads to the heating of the *B*_*p*_ coil due to the injection of a high current. For the measurement of the* ex-vivo* tissues, the temperature increase in the tissue, caused by heat transfer from the *B*_*p*_ coil, may induce undesired effects such as tissue degeneration and variation in *T*_1_ relaxation time. It is therefore important to maintain a constant tissue temperature during the measurements. The *B*_*p*_ coil system, corresponding to the 1st *B*_*p*_ dewar in Figure 1, was designed for this purpose.

For effective generation of the *B*_*p*_ at the sample region, which is beneath the base of the SQUID dewar, we adopted a pancake-type geometry for the *B*_*p*_ coil. A 700-turn copper-wire-wound pancake coil, which has an outer diameter of 132 mm and a length of 16 mm, generates a magnetic field of 60 mT at the region of the sample with a current injection of 20 A.

The *B*_*p*_ coil was mounted in a dewar, which is the green cylindrical component labeled “1st *B*_*p*_ dewar” in Figure 1, for effective cooling. Liquid nitrogen (“LN2” in Figure 1) flows continuously from the reservoir to cool the *B*_*p*_ coil. Nitrogen gas, produced by the resultant boiled liquid, then flows back out to the reservoir. The multiple layers of glass fiber in the covering plate of the dewar prevent the refrigeration of the tissue. A 3D-printed 4-mm thick thermal shield plate, having a meandering air path, was additionally placed between the sample and the covering plate of the *B*_*p*_ dewar, in order to keep the tissue temperature constant against the relatively cold surface of the dewar. The N_2_ gas was channeled continuously through the thermal shield plate, resulting in a temperature variation of ±0.3°C for thirty minutes on the top surface of the plate.

A Helmholtz coil was used for the 2nd *B*_*p*_. The field strengths were varied between 37, 62, and 122 *μ*T, which were measured at the sample space. The *B*_*m*_ and the 2nd *B*_*p*_ coils were controlled by solid state relays (SSR). However, a large amount of power is necessary to generate 1st *B*_*p*_ of approximately 60 mT, which should be switched off after a few milliseconds. Therefore, the 1st *B*_*p*_ coil was controlled by a specially constructed current-driving circuit [17], which is basically composed of a capacitor bank and an insulated gate bipolar transistor [29]. The SSRs and the current-driving circuit were remotely switched by a programmable pulse-generating board, connected by optical fibers to protect the ULF-NMR system from external electronic noise.

**Figure 2:**
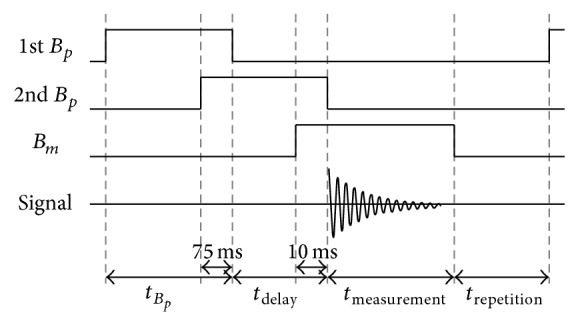
Illustration of pulse sequence for *T*_1_ experiments. The data set of *T*_1_ is obtained by changing the duration of *t*_delay_.

Figure 2 shows the pulse sequence for the *T*_1_ experiment. Initially, the 1st *B*_*p*_ is applied to form net magnetization in one direction. After the 1st *B*_*p*_ is turned off, the spins begin to relax while the 2nd *B*_*p*_ is turned on. After the time, *t*_delay_, has elapsed, the spins produce a free precession decay (FPD) signal, which is measured by the SQUID sensor. Since the degree of relaxation differs in accordance with the duration of *t*_delay_, a *T*_1_ curve is obtained by varying *t*_delay_. The following experimental parameters were used in this study (see Figure 2): *t*_*B*_*p*__ = 1 s, *t*_measurement_ = 1 s, and *t*_repetition_ = 7 s. The averaged FPD signals were measured at each time, *t*_delay_. The number of averages was mainly 20~40 depending on the sample. We measured mainly 4 points with different *t*_delay_ values for each sample in order to generate the *T*_1_ curve.

### 2.3. Analysis Method—Bayesian Analysis

A NMR signal can be expressed as the summation of *N* exponentially decaying sinusoids and Gaussian noise [30],

(1) dk=∑j=1NAjeiϕje−kΔt/τje2πikΔtfj+ɛk,k=0,…,M−1,

where *A*_*j*_ is the signal amplitude, *ϕ*_*j*_ is the phase, *τ*_*j*_ is the decay time, *f*_*j*_ is the frequency of the *j*th sinusoid, *M* is the number of data points, and *ɛ*_*k*_ is Gaussian random noise with zero mean and standard deviation, *σ*. Since this information about the NMR signal is intensively located in the beginning part of the FPD signal, it is therefore important to record the initial part of that signal.

If the SNR is low, even the initial signal is already hidden by noise, as was the case for the tissue samples investigated in this study. Therefore, we adopted the Bayesian analysis method [30], which is suitable for the analysis of small signals. The primary assumption is that the noise obtained during the data acquisition has a Gaussian distribution. After adopting the Bayesian rule, the probability becomes a function of the decay time and the resonance frequency. Consider

(2) Pd ∣ τ,f,σ,N =2πσ2N−Mλ1⋯λN  ×exp⁡⁡−∑k=0M−1dk2+∑j=1Nλjd†UEj∗/λj22σ2,

where **U** = exp⁡(−*k*Δ*t*/*τ*_*j*_)exp⁡(2*πik*Δ*tf*_*j*_), and **E** and ***λ*** are the matrices of the eigenvectors and eigenvalues of **U** ^†^ **U**, respectively. The equation of probability in [30] needs to be adjusted as illustrated in (2). Therefore, the solution is to find the maximum probability by varying two variables: the decay time and the resonance frequency. When the probability is at a maximum, the signal intensity and the phase are also determined as

(3) $$\begin{array}{c} A_{j}e^{i\phi_{j}}=\frac{{\left[E{\left(d^{†}UE\right)}^{*}\right]}_{j}}{\lambda_{j}}. \end{array}$$

Since the resonance frequency can be determined by the FFT of the relatively larger signal or the measurement of the *B*_*m*_ strength, the variable of the resonance frequency is additionally reduced. Therefore, more precise information on the signal intensity could be obtained from the FPD signal. During the analysis of the acquired data, nondecaying terms such as the higher harmonics of the AC line noise and/or the additional noise were also considered by adding these terms to the equations, whereas only one decaying term corresponding to the FPD signal was used.

## 3. Results

**Figure 3:**
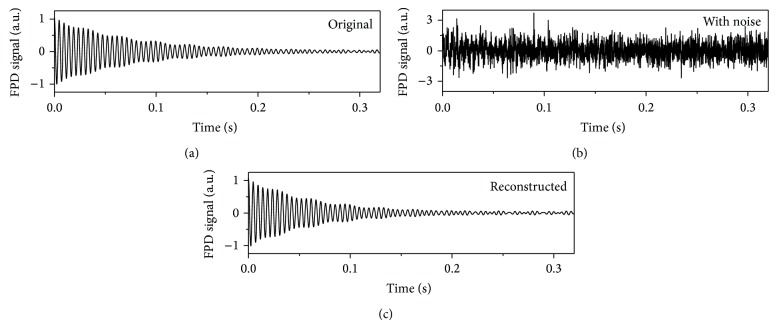
Simulation of Bayesian analysis: (a) the simulated FPD signal, (b) the mixed signal with Gaussian random noise, and (c) the reconstructed signal from the noisy signal plotted in (b). The simulated FPD signal was built on assumptions of *A*_*j*_ = 1, *ϕ*_*j*_ = 0, *τ*_*j*_ = 0.08, and *f*_*j*_ = 211 in (1). The nondecaying signals were also added as electronic noise, which had the same amplitude of 0.02 and frequencies of 180 and 240 Hz. The simulated FPD signal was mixed with Gaussian random noise, which had a standard deviation of 0.8. After Bayesian analysis, the reconstructed signal had the decaying components *A*_*j*_ = 1.031, *ϕ*_*j*_ = 0.011, *τ*_*j*_ = 0.066, and *f*_*j*_ = 211. The nondecaying components of the reconstructed signal were composed of the amplitudes and phases of *A*_180_ = 0.026, *A*_240_ = 0.027 and *ϕ*_180_ = −0.857, *ϕ*_240_ = 0.314, respectively.

To estimate *T*_1_ relaxation times, the variation in the FPD signal amplitudes was measured as a function of the time duration, *t*_delay_. Each data set was fitted using the following equation:

(4) $$\begin{array}{c} f\left(t\right)=\alpha \exp\left(-\frac{t_{\text{delay}}}{T_{1}}\right), \end{array}$$

where *α* is the amplitude and *T*_1_ is the longitudinal relaxation time. The additional constant to represent the base-offset was not included, because the Bayesian analysis described above discriminates NMR signals from noisy backgrounds well (see Figure 3 and/or Supplementary Information in Supplementary Material available online at http://dx.doi.org/10.1155/2014/385428).

**Figure 4:**
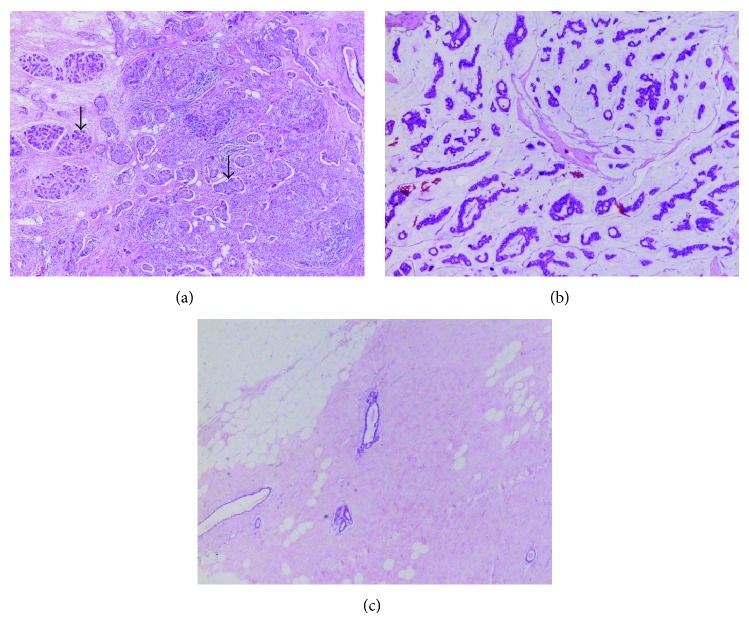
Photomicrographs of (a) the invasive ductal carcinoma sample (Specimen number S13-10), (b) the mucinous carcinoma sample (Specimen number S13-09), and (c) normal breast tissue (Specimen number S13-09). The arrows on the right- and left-hand sides of the picture in (a) represent irregularly infiltrating epithelia cell clusters and a well-organized normal breast lobule, respectively. Each specimen was stained with hematoxylin-eosin and observed at ×40 magnification.

Table 1 summarizes the pathologic characteristics, fraction of cancerous cells in each tumor and normal tissue (the rest of the tissue is filled with normal cells), and the measured *T*_1_ values at three magnetic fields of the thirteen pairs of breast tissue specimens, which exhibited FPD signals. The examples of *T*_1_ fitted curves are shown in the Supplementary Information section. In addition, two *T*_1_ values could not be estimated using the fitting equation, because the number of data points obtained by the FPD signal were insufficient. In those cases, the baseline of the FPD signal fluctuates and is discontinued. Most tumor specimens were composed of cancer cells, surrounding desmoplastic stroma, and some normal epithelial cells. For example, the invasive ductal carcinoma (Specimen number S13-10) plotted in Figure 4(a) shows irregularly infiltrating epithelia cell clusters and a well-organized normal breast lobule, which are denoted by the arrows on the right- and left-hand sides of the picture, respectively. The mucinous carcinoma specimen, however, had only cancer cells and mucin with no normal tissue. The mucinous carcinoma sample (Specimen number S13-09), plotted in Figure 4(b), showed a few clusters of malignant epithelial cells in the background of the mucin pool, whereas the normal breast tissue was composed of fibrotic stroma and a few epithelial cells (see Figure 4(c)). Among the four cases receiving preoperative chemotherapy, one case (Specimen number S13-02) responded to treatment and the majority of the tumor tissue was replaced with fibrotic stroma.

Since the tumor tissues contain partial cancers, the *T*_1_ values estimated from the fitting do not yield meaningful information about the *T*_1_ contrast unless properly analyzed. For correct estimation of the *T*_1_ contrast between cancer and normal tissue, the cancer volume ratio must be considered as part of the analysis. The model devised by Zimmerman and Brittin [27] describes the relaxation phenomenon in a system of multiple phases. According to this model, the exchange rate between the two media with different *T*_1_ relaxation times strongly influences the *T*_1_ relaxation process. We presume that the exchange rate of the water molecules between normal and cancer cells in a tumor tissue is reasonably rapid compared with the relaxation rate 1/*T*_1_. Then, a single exponential decay with the average relaxation rate, 1/*T*_1*T*_ = *ρ*_*C*_/*T*_1*C*_ + *ρ*_*N*_/*T*_1*N*_, will be obtained, in which *T*_1*C*_ and *T*_1*N*_ are the *T*_1_ relaxation times of the cancer and normal cells, respectively. The *ρ*_*C*_ and *ρ*_*N*_ are volume ratios of the cancer and normal cells in the tissue. The ratio between *T*_1*N*_ and *T*_1*T*_, that is, *T*_1*N*_/*T*_1*T*_, of each tumor tissue should show a linear dependence on the *ρ*_*C*_ and the coefficient is the *T*_1_ contrast, *δ*, between the cancer and normal cells as

(5) $$\begin{array}{c} \frac{T_{1N}}{T_{1T}}=1+\rho_{C}\left(\frac{T_{1N}}{T_{1C}}-1\right)=1+\rho_{C}\delta . \end{array}$$

**Figure 5:**
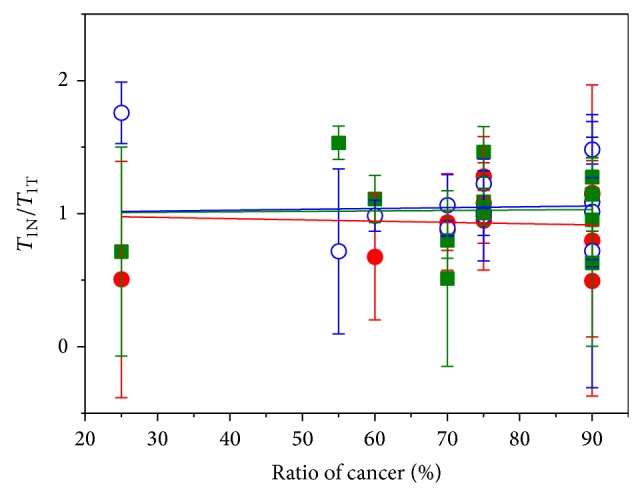
The *T*_1_ ratio between the tumor (T) and the normal (N) tissue as a function of the cancer ratio. Since the normal tissue in specimen S13-05 was 5% cancerous, the surface ratio of the cancer of this specimen was denoted as 55%. The (red) closed circles, (blue) open circles, and (green) closed squares represent the data obtained at 122, 62, and 37 *μ*T, respectively. The error bars indicate the standard errors. The lines are linear fits to data obtained at each field.

Figure 5 shows the *T*_1_ ratios between the tumor and the healthy tissue (*T*_1*N*_/*T*_1*T*_) as a function of the relative cancer volume, *ρ*_*C*_. The (red) closed circles, (blue) open circles, and (green) closed squares represent the data obtained at 122, 62, and 37 *μ*T, respectively. Apparently, the *T*_1_ relaxation time has a weak dependency on *ρ*_*C*_, and the *T*_1_ contrast, *δ*, exhibits a weak variance as the magnetic field increases. The estimated *δ* is 3.3 × 10^−4^ at 37 *μ*T, 6.3 × 10^−4^ at 62 *μ*T, and −9.4 × 10^−4^ at 122 *μ*T, which do not appear to be meaningful compared with standard deviations of the data. Possible reasons for this will be discussed below.

**Figure 6:**
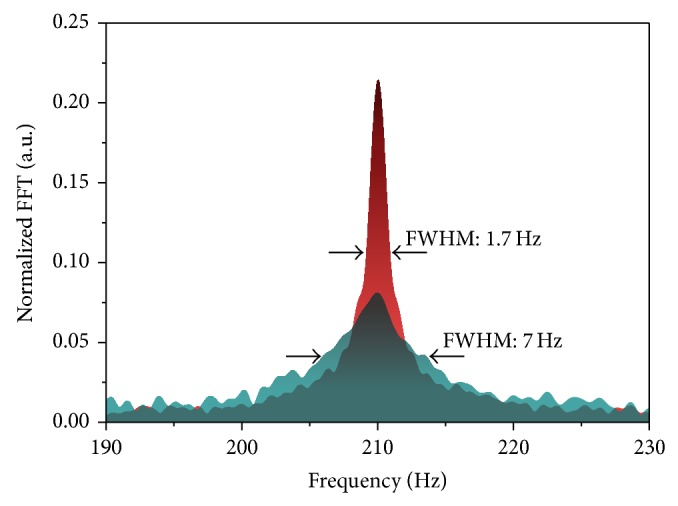
The normalized spectra for the tumor tissues of S13-09 (red) and S13-10 (green) obtained by the experimental conditions of *t*_delay_ = 10 ms and 2nd *B*_*p*_ = 62 *μ*T. The spectra were normalized with each spectrum area.

From the mucinous carcinoma tissue (Specimen number S13-09), a clearly distinguishable NMR signal was measured, as shown in Figure 6. The NMR spectrum has a full-width half-maximum (FWHM) of 1.7 Hz, which is about one quarter of the FWHMs of the other tissue, which are typically 7 Hz. As a result, *δ* is higher than 4. As in the case of high-field MRI [31], mucinous carcinoma tissue can be identified unambiguously in low field MRI via *T*_1_ or *T*_2_ weighted imaging methods.

## 4. Discussion

In the multiphases relaxation model [27], (5) is based on the assumption that water molecules in cancer and normal cells can be exchanged rapidly compared with the relaxation rates of water molecules belonging to separate phases in the system. If this assumption is not valid, the *T*_1_ relaxation of tissue should exhibit multiple exponentials. The *T*_1_ values displayed in Table 1 are mostly <100 ms. Therefore, the exchange rate between water molecules in cancer and normal cells should be at least 10 s^−1^ in order for (5) to be applied. Such an exchange rate might not be a realistic value. We speculate that cancer cells are still isolated from normal cells for longer periods than the time scale of 100 ms. In this case, multiple exponential fitting of the decay curve should be required to correctly analyze tissue samples having a cancer ratio in the range of 20~80%. The relaxometry of a single (or finite) voxel in the MR imaging of tumor tissue will therefore be a better approach to distinguishing cancer volumes from normal cells when they are partially mixed, as in our case.

Experimental attempts to obtain the optimal *T*_1_ contrast between cancerous and healthy tissue can be supported by microscopic physiology studies on cancer cells. As the Warburg effect states [32], cancer cells produce mostly lactate at the end of their metabolic cycles, while normal cells mostly produce ATPs as an energy source. The differentiated by-products of the metabolic cycle may involve different influences on the relaxation mechanism of water molecules near to those chemicals. For instance, the relaxation rate of water molecules in protein is affected by the molecular weight of the protein [33]. Justification for applying a low magnetic field rather than a high field will finally come only after a better understanding of the relaxation process between by-products of metabolites and water molecules in cells. To this end, further physiology-orientated studies at low magnetic fields should be conducted in the future.

The limitations in the interpretation of our results exist in the present work. First, the measured *T*_1_ values of several tissues in Table 1 exhibit rather large variation in the estimated errors. Currently, we suspect that the long averaging time, typically 20 min. for a single *T*_1_ measurement, may cause thermally induced problems such as slight variance or degradation of *B*_*p*_ due to the heated coil. That is reasonable if the circulation of LN2 is not smooth in an instant due to the local heating of *B*_*p*_ coil, although there is no heat dissipation at the sample position by using a specially designed *B*_*p*_ coil system. Therefore, it is definitely required to reduce the averaging time in order to minimize the unwanted thermal effects during the measurements. Second, the comparison between benign and malignant tissues is missing. In this work, malignant tissues are compared only with normal tissues. Third, the fifteen pairs of tissues investigated in this work may not seem sufficient for a reliable statistical analysis. Particularly, the result obtained from the mucinous tissue should be confirmed by investigating more tissues, although mucinous type is rare. Finally, the collected tissues were frozen and preserved in LN2 dewar before the *T*_1_ measurements. The freezing and thawing process may cause a damage in the tissues. In future study, a method of preforming relaxation measurement before freezing is needed to be devised.

In terms of SNR, we believe two points can be improved, which will render single voxel relaxometry of breast cancer feasible. First, the environmental noise of our system, including SQUID, was measured to be 2.3 fT/Hz^1/2^, and this value is relatively higher than the other group's noise environment below 1 fT/Hz^1/2^ [13, 25]. Second, the SNR will increase if the *B*_*p*_ strength is increased by optimizing the coil system. Currently, it was limited to a maximum of 60 mT due to heat dissipation from the coil.

## 5. Conclusions

In this study, we performed *T*_1_ relaxation measurements of* ex-vivo* breast cancer tissue samples in order to verify the feasibility of confirmative MR imaging of breast cancer. Since the volumes of the investigated tissues were small, improvements on our ULF-NMR setup and signal processing approach were required. A specially designed *B*_*p*_ coil system, including liquid nitrogen cooling and thermal isolation, produces a reliable high field without heat dissipation at the sample position. In addition, noise elimination using Bayesian statistics enabled us to overcome a relatively small SNR. In spite of those efforts, the *T*_1_ relaxometry, measured at 37, 62, and 122 *μ*T, failed to exhibit a meaningful fingerprint expected at low magnetic fields. At this moment, however, we can not conclude that the absence of the fingerprint is an intrinsic characteristic of ductal- and lobular-type breast cancer tissues, owing to the remaining limitations discussed in the previous section. Therefore, further improvements of the systems and methods are required to obtain valuable outputs. In contrast, mucinous cancer showed a reliable enhancement in *T*_1_ contrast, which would be a useful application of ULF-MRI. This work is the first experimental approach toward the discrimination of breast cancer tissues by *T*_1_ contrasts at low fields. The equipment and methodologies that we developed in this study are believed to be a useful guide to future work.

## Supplementary Material

Additional experimental results and information not shown in the text are provided in the Supplementary Information section: an example of Bayesian analysis applied to an actual signal, the description about the removal of the base-offset in the fitting function, and some examples of T_1_ fitted curves.

## Figures and Tables

**Table 1:** Analyzed and measured parameters.

Specimen number	Type	Cancer %		*T*_1_ at 122 *μ*T (ms)		*T*_1_ at 62 *μ*T (ms)		*T*_1_ at 37 *μ*T (ms)	
		Tumor tissue	Normal tissue	Tumor tissue	Normal tissue	Tumor tissue	Normal tissue	Tumor tissue	Normal tissue
S13-01	Ductal	90	0	60.4 ± 45.7	48.2 ± 26.4	84.1 ± 21.9	91.4 ± 42.2	109.5 ± 42.9	69 ± 3
S13-02^a^	Ductal	25	0	161.1 ± 53.2	81.3 ± 24.7	53.5 ± 10.8	94.1 ± 33.2	79 ± 35.2	56.4 ± 19.3
S13-03	Ductal	75	0	68.7 ± 9.9	74 ± 38.5	63.4 ± 2.9	62.3 ± 20.5	66.8 ± 13.7	97.7 ± 18.4
S13-04^a^	Lobular	60	0	81 ± 20.1	54.6 ± 11	61.8 ± 5	60.8 ± 5	59.5 ± 8.9	66 ± 8.4
S13-05^a^	Ductal + lobular	60	5	100.1 ± 7.4	—	121 ± 53.3	86.6 ± 5	68.5 ± 10.4	105 ± 12.4
S13-06	Ductal	70	0	72.2 ± 22.8	67.3 ± 7.5	63.6 ± 13.8	67.6 ± 8.3	79.4 ± 5.2	63.4 ± 5.3
S13-07	Ductal	75	0	79.2 ± 5.8	101.3 ± 11.2	73.6 ± 4.8	74.4 ± 12.3	68.2 ± 4.8	74.2 ± 6.4
S13-08	Ductal	90	0	169.2 ± 14.7	83.3 ± 15.6	81.9 ± 28.3	82.9 ± 9.3	64.1 ± 18.4	61.2 ± 12.8
S13-09	Mucinous	100	0	347.7 ± 24.9	77.5 ± 26.9	358.6 ± 25.8	84.7 ± 13.4	—	46.3 ± 5.6
S13-10	Ductal	75	0	67 ± 3	63.5 ± 9.7	68.9 ± 2.7	84.4 ± 19	65.6 ± 2.4	65.9 ± 5.6
S13-11^a^	Ductal	90	0	86.1 ± 7.9	98.4 ± 29.8	84 ± 6.1	60.3 ± 44.3	76.5 ± 1.3	87.6 ± 27.8
S13-12	Ductal	70	0	115 ± 11.7	100.6 ± 8.5	91.2 ± 3.3	81.2 ± 2.7	49.8 ± 7.1	25.5 ± 7.8
S13-13	Ductal	90	0	65.5 ± 0.3	75.8 ± 21.2	52.2 ± 15.4	77.3 ± 8.1	64 ± 3.1	81.6 ± 14.5

^a^The preoperative chemotherapy was performed.
