# Supplementary material for: T 1 Relaxation Measurement of Ex-Vivo Breast Cancer Tissues at Ultralow Magnetic Fields
Source: Biomed Res Int. 2015 Jan 29;2015:385428. doi: 10.1155/2015/385428 (PMC4326347; doi:10.1155/2015/385428)
Supplement: Supplementary file 1 — Additional experimental results and information not shown in the text are provided in the Supplementary Information section: an example of Bayesian analysis applied to an actual signal, the description about the removal of the base-offset in the fitting function, and some examples of T1 fitted curves. [file 385428.f1.zip › SI_Cancer.pdf]

Supplementary Information to the Manuscript "T_1 relaxation measurement of *ex-vivo* breast cancer tissues at ultralow magnetic fields"

Seong-Joo Lee[1*], Jeong Hyun Shim[1*], Kiwoong Kim[1,2†], Seong-min Hwang[1], Kwon Kyu Yu[1], Sanghyun Lim[1,2], Jae Ho Han[3], Hyunee Yim[3], Jang-Hee Kim[3], Yong Sik Jung[4], and Ku Sang Kim[4]

[1] Center for Biosignals, Korea Research Institute of Standards and Science (KRISS), 267, Gajeong-ro, Yuseong-gu, Daejeon 305-340, Republic of Korea

[2] Department of Medical Physics, University of Science and Technology (UST), 217, Gajeong-ro, Yuseong-gu, Daejeon 305-333, Republic of Korea

[3] Department of Pathology, Ajou University School of Medicine, 164, World Cup-ro, Yeongtong-gu, Suwon 443-380, Republic of Korea

[4] Department of Surgery, Ajou University School of Medicine, 164, World Cup-ro, Yeongtong-gu, Suwon 443-380, Republic of Korea

[*]These authors contributed equally to this work.
[†]Correspondence to: Kiwoong Kim, Ph.D., Center for Biosignals, KRISS, Republic of Korea. Email: kwkim@kriss.re.kr

1. Comparison of actual FPD signals before and after the Bayesian analysis

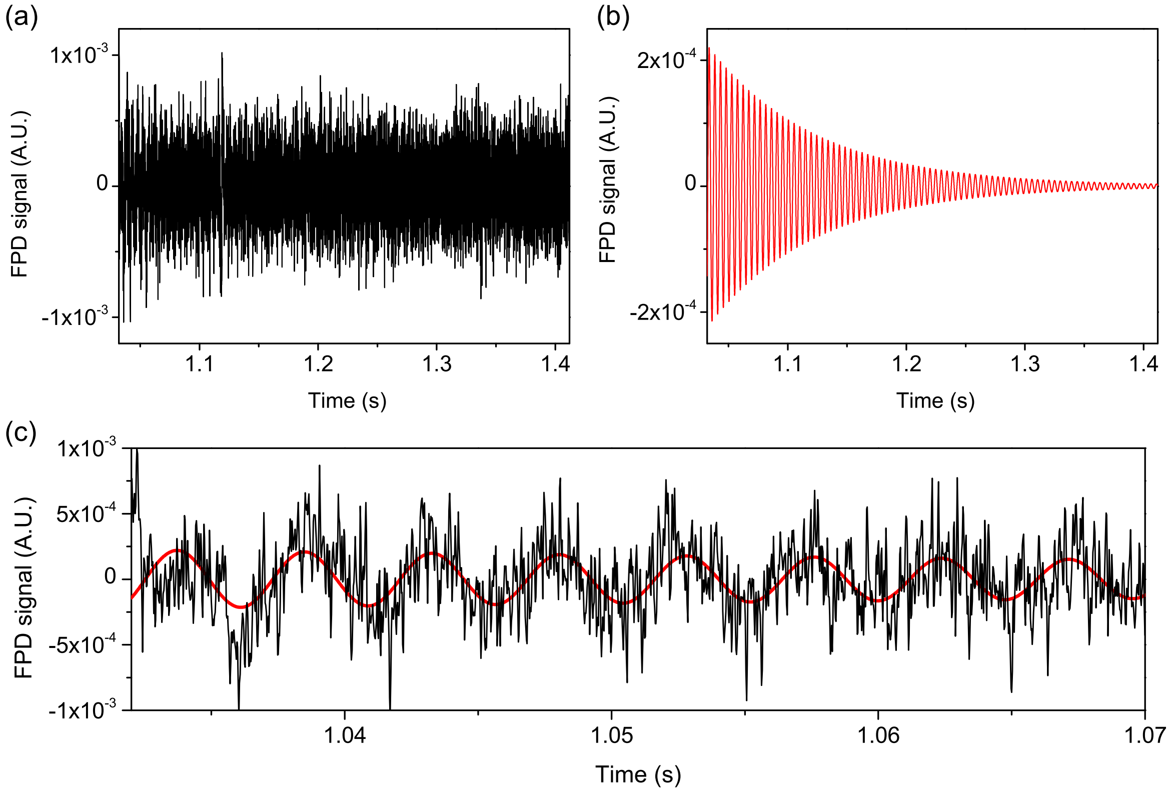

Figure SI.1: Example of actual FPD signals before and after the Bayesian analysis: (a) The FPD signal of the normal tissue (Specimen No. S13-01) obtained by the experimental conditions of $t_{\mathrm{delay}} = 10$ ms and $2^{\mathrm{nd}}B_{\mathrm{p}} = 37\ \mu$T, (b) the reconstructed FPD signal from the actual FPD signal plotted in (a), and (c) the magnified view of the graph for both signals. The black and red solid lines represent the actual and reconstructed FPD signals, respectively. The Bayesian analysis described above discriminates NMR signals from noisy backgrounds well.

2. Omission of offset parameter from T_1 fit function

In the T_1 fitting function, Equation (4) in the main text, we excluded the offset parameter. If the offset must be included in the analysis, it is owing to two possible reasons. One is that the $2^{\text{nd}} B_{\text{p}}$ is non-zero during the time t_{delay}. This will produce a non-zero nuclear magnetization even after a long t_{delay}. In our experiments, however, the $2^{\text{nd}} B_{\text{p}}$ values were 37, 62, and 122 μT, thus the ratio of the field intensities between $1^{\text{st}} B_{\text{p}}$ and $2^{\text{nd}} B_{\text{p}}$ varied from 0.06% to 0.2%, which are still negligible contributions.

The other is related to the signal-to-noise ratio (SNR). Figure 3 and Figure SI.1 shows the result of the Bayesian analysis (BA). The SNRs of the FPD signals before the BA were approximately 1 in both cases. The simulation result of BA (Figure 3), however, showed that the reconstructed amplitude exhibited approximately 3% error compared with the original amplitude. This means that, in our analysis, the base-offset could have been included but only in a baseline fluctuation of less than 3%. Thereby, the offset parameter in the fitting function can be omitted in the analysis. Conclusively, the exponential decay without base offset was reasonably set for efficient fitting analysis.

3. Examples of T_1 fitted curves for each specimen

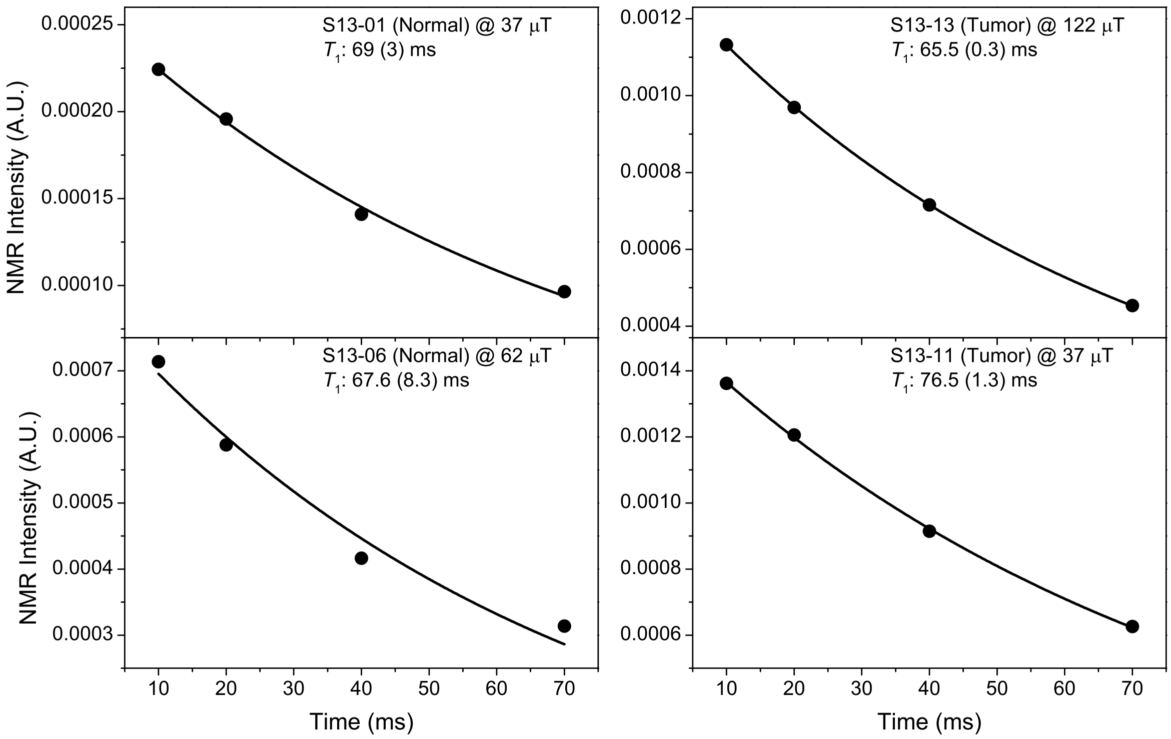

Figure SI.2: Samples of T_1 fitted curves for each specimen. Information on the specimen number, tissue type, $2^{nd}B_p$ strength, and the estimated T_1 value were specified in each curve. The value within parentheses represents the standard error of the estimated T_1 value. The curves are randomly chosen under the condition that the standard error was below 10 ms (see Table 1). The T_1 fitted curves of the normal and tumor tissue samples are plotted on the left- and right-hand sides, respectively.

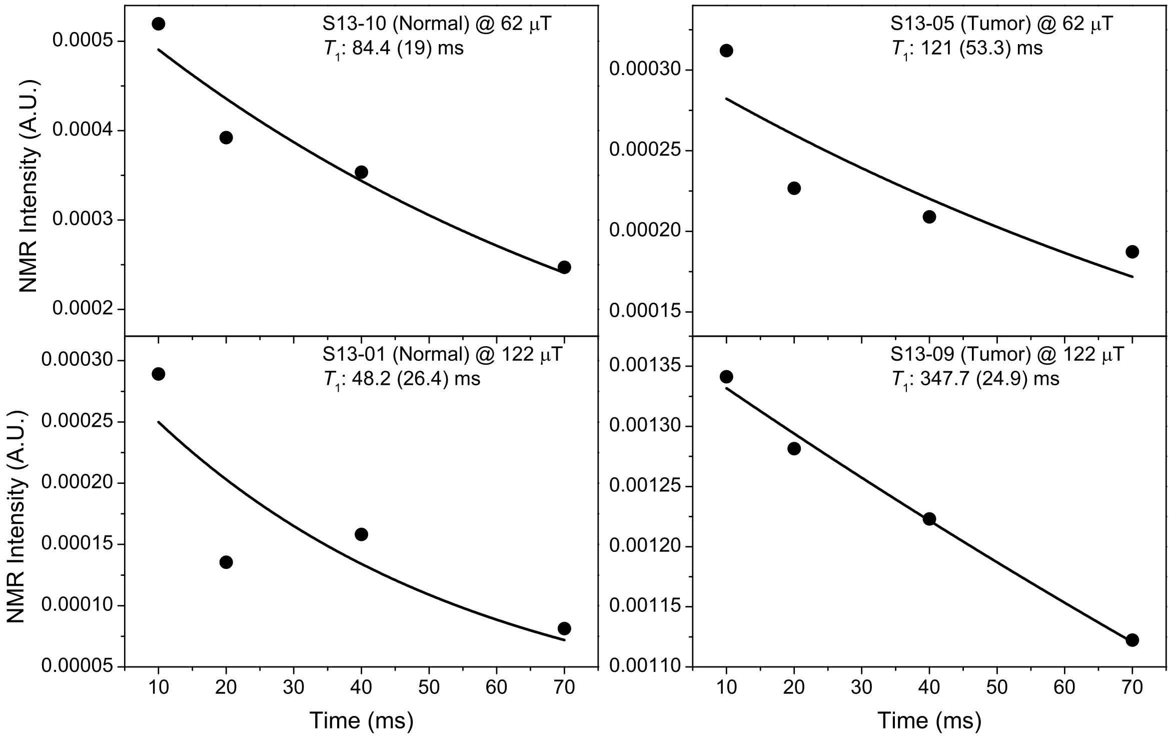

Figure SI.3: Samples of T_1 fitted curves for each specimen. Information on the specimen number, tissue type, $2^{\mathrm{nd}} B_{\mathrm{p}}$ strength, and the estimated T_1 value were specified in each curve. The value within parentheses represents the standard error of the estimated T_1 value. The curves are randomly chosen under the condition that the standard error was above 10 ms (see Table 1). The T_1 fitted curves of the normal and tumor tissue samples are plotted on the left- and right-hand sides, respectively.
